# Methodology for the objective and rapid determination of the performance criteria of an NH_3_-NaSCN absorption refrigeration system

**DOI:** 10.1016/j.mex.2022.101918

**Published:** 2022-11-08

**Authors:** G.R.H. Ngock, J.G. Tamba, S. Essiane Ndjakomo, F.L. Djanna Koffi, M. Ndame

**Affiliations:** Laboratory of Technology and Applied Sciences, University of Douala, Cameroon

**Keywords:** Characteristic matrix, Concentration, Easy to code, Simulation platform

## Abstract

This work is a computer-aided methodological description for the rapid and objective analysis of the performance criteria of an absorption refrigerator. It can be used as a tool for a simulation platform to improve the parameters for the optimal operation of the present system. Sometimes, some of the obstacles of the original method related to the analysis of the performance criteria of absorption machines may come from the number of equations and the complexity of the calculation of NH3 mass fraction of NH_3_-NaSCN solution. The method consists of a characteristic matrix that allows to quickly determine the NH_3_ mass fraction of NH_3_-NaSCN solution in relation to the performance criteria: COP (coefficient of performance), ECOP (exergetic efficiency) and CR (circulation ratio).This could facilitate the algorithm and the direct calculation of NH_3_ mass fraction of NH_3_-NaSCN solution in contrast to the original model. For this reason, an easily reproducible flow chart has been proposed.

• Transformation of complex mathematical models into easy to code models.
• Elaboration of a characteristic matrix for the determination of concentrations.
• Development of a platform for the simulation of performance criteria.

## Graphical abstract

**Figure:**
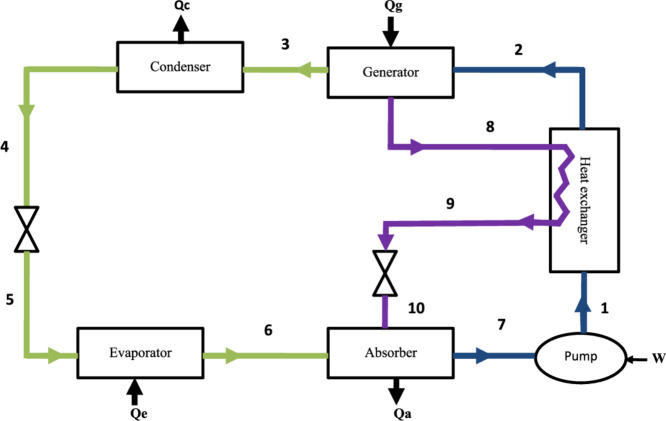

Specifications table

**Table:** 

Subject Area:	*Energy*
More specific subject area:	*Refrigeration absorption*
Method name:	*Minimised method*
Name and reference of original method:	*Method's name: Refenrence*:Sun *D.W.Comparaison of performances of NH3-H_2_O, NH_3_-LiNO_3,_ NH3-NaSCN.Energy Conversion and Mangemement (1998) 39:357-368)*
Resource availability:	*No* resource available

## Presentation of the fluid used

**Fig. 1:**
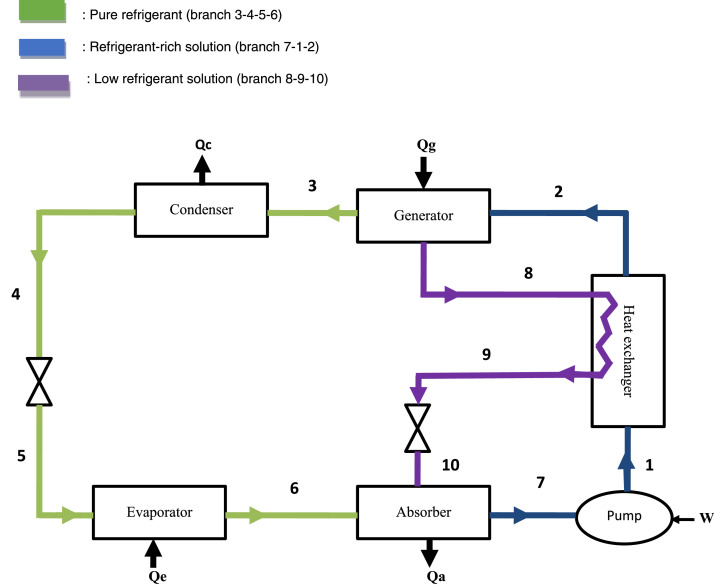
Scheme of the model [2].

This system uses the NH_3_-NaSCN pair, which can generate two chemical compounds during operation: the refrigerant (NH_3_), capable of being evaporated at low temperatures [1], and the solution (NH_3_-NaSCN), which may be rich or poor in NH_3._ The refrigerant (NH_3_) can be in the vapour state at points (3) and (6), or in the liquid state at points (4) and (5) (Fig. 1). The solution

(NH_3_-NaSCN) can be refrigerant-lean as it flows through the 8-9-10 branch and refrigerant-rich on the 7-1-2 branch. The legend of the nature of the fluids flowing in each branch of the system (Fig. 1) is presented using the colour code below:

## System operation

The operating principle is as follows: The pump (1) propels the fluid mixture into the boiler (2). This mixture under goes desorption, sending the refrigerant to the condenser (3) and the refrigerant-lean solution to the absorber. The refrigerant from the condenser passes through the evaporator (6) and into the absorber. The mixture from the absorber (7) is directed to the pump and the cycle starts again.

## Mathematical modelling

This section presents two approaches: an original classical model and a proposed new model based on a one-line variable characteristic matrix. The original model regularly used by researchers is based on conservation laws, assisted by correlations of thermodynamic properties from two different approaches, namely that of sun [1] for NH_3_ and that of Ferreira [3] for the NaSCN solution. This generally makes the direct calculation of NH_3_ mass fraction of NH_3_-NaSCN solution more complex and difficult to code. The new model proposed in this work is obtained by combining the correlations of Sun [1] and Ferreira [3], in order to obtain a variable one-line characteristic matrix. This combination allows to reduce some intermediate equations of the original model and to find a one-line variable matrix that allows to calculate directly the NH_3_ mass fraction of NH_3_-NaSCN solution at any point of the system. The matrix obtained from the two different correlations is the main point of the new proposed method, which also participates in the calculation of the performance criteria, namely the COP (coefficient of performance), the ECOP (exergetic efficiency) and the CR (circulation ratio) of the present system. It is therefore the basic element for reducing the intermediate equations of the original model, and for calculating the NH_3_ mass fraction of NH_3_-NaSCN solution which could be a major obstacle of the original algorithm that hinders the coding to calculate the performance parameters.

### Original classical model

This section is devoted to the mathematical representation of the system's operation on the basis of real physical phenomena . The original methodology is based on the Sun model [1] used by several researchers in the literature. This model is a combination of several equations, using two tools: the conservation laws and the thermodynamic properties of the fluid. This model is based on the following simplifying assumptions:

• The expansion is isenthalpic on the refrigerant and solution side;
• The refrigerant leaving the condenser and evaporator is saturated;
• Internal dissipation losses are negligible;
• The solutions leaving the absorber and the generator are saturated;
• The refrigerant leaving the generator is superheated;
• The liquid in the evaporator is completely evaporated and only vapour passes to the absorber.

Eqs. (1)–(26) of the conservation law are respectively applied as follows:

➤ Generator
  (1) $${\dot{m}}_{2}={\dot{m}}_{8}+{\dot{m}}_{3}$$
  (2) $${\dot{m}}_{2}x_{2}={\dot{m}}_{8}x_{8}+{\dot{m}}_{3}$$
  (3) $${\dot{Q}}_{g}={\dot{m}}_{3}h_{3}+{\dot{m}}_{8}h_{8}-{\dot{m}}_{2}h_{2}$$

Where ${\dot{m}}_{i,}\;X_{i}$ and $h_{i}\left(1\leq i\leq 10\right)$ are respectively the mass flow rate, NH_3_ mass fraction of NH_3_-NaSCN solution and enthalpy at point *i*; Q_g_ thermal power of the generator.

➤ Evaporator
  (4) $${\dot{m}}_{5}={\dot{m}}_{6}$$
  (5) $$x_{5}=x_{6}$$
  (6) $${\dot{Q}}_{e}={\dot{m}}_{6}h_{6}-{\dot{m}}_{5}h_{5}$$

with ${\dot{Q}}_{e}$ thermal power of the evaporator

➤ Condenser
  (7) $${\dot{m}}_{3}={\dot{m}}_{4}$$
  (8) $$x_{3}=x_{4}$$
  (9) $${\dot{Q}}_{C}={\dot{m}}_{3}h_{3}-{\dot{m}}_{4}h_{4}$$

with ${\dot{Q}}_{c}$ thermal power of the condenser.

➤ Absorber
  (10) $${\dot{m}}_{7}={\dot{m}}_{6}+{\dot{m}}_{10}$$
  (11) $${\dot{m}}_{7}x_{7}={\dot{m}}_{6}+{\dot{m}}_{10}x_{10}$$
  (12) $${\dot{Q}}_{a}={\dot{m}}_{6}h_{6}+{\dot{m}}_{10}h_{10}-{\dot{m}}_{7}h_{7}$$

with ${\dot{Q}}_{a}$ thermal power of the evaporator.

➤ Pump
  (13) $${\dot{m}}_{1}={\dot{m}}_{7}$$
  (14) $${\dot{X}}_{1}={\dot{X}}_{7}$$
  (15) $$\dot{W}=\frac{{\dot{m}}_{1}}{\rho }\left(P_{1}-P_{7}\right)$$
  (16) $$P_{1}-P_{7}=\left(h_{1}-h_{7}\right)\rho$$

With $\dot{W}$ power of the pump and ρ the density.

➤ Heat exchanger
  (17) $${\dot{m}}_{8}={\dot{m}}_{9}$$
  (18) $${\dot{m}}_{1}={\dot{m}}_{2}$$
  (19) $$ef=\frac{T_{8}-T_{9}}{T_{8}-T_{1}}$$
  (20) $${\dot{Q}}_{\text{ex}}={\dot{m}}_{8}\left(h_{8}-h_{9}\right)={\dot{m}}_{2}\left(h_{2}-h_{1}\right)$$

$T_{i}$ the temperatureat the *i* point,(1≤i≤10) ; *ef* efficiency of the exchanger, and ${\dot{Q}}_{\text{ex}}$ the power of the exchanger.

Expansion valve on the refrigerant side

(21) $${\dot{m}}_{4}={\dot{m}}_{5}$$

(22) $$x_{4}=x_{5}$$

Expansion valve on the solution side

(23) $${\dot{m}}_{9}={\dot{m}}_{10}$$

(24) $$x_{9}=x_{10}$$

From Eqs. (1) and (2), the following equations can be derived:

(25) $${\dot{m}}_{8}=\frac{1-X_{2}}{X_{2}-X_{8}}{\dot{m}}_{3}$$

(26) $${\dot{m}}_{2}=\frac{1-X_{8}}{X_{2}-X_{8}}{\dot{m}}_{3}$$

The Eqs. (27)–(29) related to the thermodynamic properties of the refrigerant (NH_3_), both in the liquid and vapour state, were mentioned by Sun [1]:

➤ NH_3_ refrigerant

Pressure versus temperature at any point in the system:

(27) $$P\left(T\right)=10^{3}\sum_{i=0}^{6}a_{i}{\left(T-273,15\right)}^{i}$$

For the liquid refrigerant, the temperature versus enthalpy at any point is:

(28) $$\text{hl}\left(T\right)=10^{3}\sum_{i=0}^{6}b_{i}{\left(T-273,15\right)}^{i}$$

For the vapor refrigerant, the temperature as a function of enthalpy at any point is:

(29) $$\text{hv}\left(T\right)=10^{3}\sum_{i=0}^{6}c_{i}{\left(T-273,15\right)}^{i}$$

Table 1 shows the coefficients a_i_, b_i_ and c_i_:

**Table 1:** Table of coefficients.

i	a_i_Eq. (27)	b_i_Eq. (28)	c_i_Eq. (29)
0	4.2871 e-1	1.9879 e2	1.4633 e3
1	1.6001 e-2	4.4644 e0	1.2839 e0
2	2.3652 e-4	6.2790 e-3	-1.1501 e-2
3	1.6132 e-6	1.4591 e-4	-2.1523 e-4
4	2.4303 e-9	-1.5262 e-6	1.9055 e-6
5	-1.2494 e-13	-1.8069 e-8	2.5608 e-8
6	1.2741e-13	-1.9054 e-10	-2.5964 e-10
Standard error	1.6e-3	8.5626 e0	1.059 e1
Mean deviation	1.252e-2	5.566 e-3	3.679 e-3

The Eqs. (30)–(41) related to the thermodynamic properties of the NH_3_-NaSCN solution were mentioned by Ferreira [3].

➤ The NH_3_-N_a_SCN solution

The pressure and temperature of the solution are related by:

(30) $$\text{lnP}=A+\frac{B}{T}$$

(31) AP=15.7266−0.298628X

(32) $$\text{BP}=-2548.6-2621.92{\left(1-X\right)}^{3}$$

The enthalpy here is a two-variable function defined by:

(33) h(T,X)=A+B(T−273.15)+C(T−273.15)2+D(T−273.15)3

(34) A=79.72−1072X+1287.9.X2−3.5137X3

(35) B=2.4081−2.2814.X+7.9291×2−3.5137X3

(36) C=10−2(1.255X−3X2+3.06X3)

(37) D=10−5(−3.33X+10X2−3.33X3)

The same holds for density:

(38) ρ(T,X)=A+B(T−273.15)+C(T−273.15)2

(39) A=1707.519−2400.4248X+2256.5083X2−930.063X3

(40) B=3.6341X+5.4552X2−3.164X3

(41) C=10−3(5.1X−3.6X2+5.4.X3)

The parameters characterizing the performance criteria of the system are given in the Eqs. (42)–(44).

  • The circulation ratio (CR):

This is the size indicator of the machine. The objective is to reduce it further to maximise the refrigerant. It is defined by [1]:

(42) $$\text{CR}=\frac{{\dot{m}}_{2}}{{\dot{m}}_{3}}=\frac{1-X_{8}}{X_{2}-X_{8}}$$

  • The coefficient of performance (COP)

This is the indicator characterising the amount of performance of the system. The objective is to maximise it to improve the amount of energy in the system. It is defined by [1]:

(43) $$\text{COP}=\frac{{\dot{Q}}_{e}}{{\dot{Q}}_{g}+W}$$

  • Exergetic efficiency (ECOP)

This is the indicator characterising the performance quality of the system. The objective is to maximise it also to improve the quality of performance. It is defined by [4]:

(44) $$\text{ECOP}=\frac{{\dot{Q}}_{e}\left(1-\frac{T_{0}}{T_{e}}\right)}{\dot{W}+{\dot{Q}}_{g}\left(1-\frac{T_{0}}{T_{g}}\right)}$$

with T_0_ room temperature, T_e_ evaporating temperature and T_g_ generator temperature.

### Model of the characteristic matrix

This part consists in transforming the original global model usually elaborated by the researchers, into a reduced model easily transformable into a simple scientific program whatever the software used for coding. In this section, we develop this easily implementable reduced model based on the original model and the assumptions already mentioned above. However, the determination of the NH_3_ mass fraction of NH_3_-NaSCN solution noted *X_i_* is very crucial for the fast calculation of the performance parameters: CR, COP and ECOP. The new model is based on the determination of the characteristic matrix of *X_i_*. The simplification of the original model starts by equalizing the pressure between the correlations (27) and (30) associated with Table 1 and then substituting the empirical formulas (31) and (32). This equality can be established by simultaneously choosing two portions of the same pressure level based on the empirical formula of Sun [1] for NH_3_, and Ferreira [3] for NaSCN. This provides the characteristic matrix *Mi* at a point i for any temperature *T_i_* of the system. This matrix is given by Eq. (45) below:

(45) $$M_{i}=[\frac{2621.92}{T_{i}+273.15},\;-\frac{3*2621.92}{T_{7}+273.15},\;\frac{3*2621.92}{T_{7}+272.851372},\;\frac{-5154.8434}{T_{7}+273.15\log\left(P_{i}\right)}]$$

The existence of the characteristic matrix, which contributes to the reduction of certain intermediate equations, can also be used to solve all the equations related to the variable *X_i_*. The coefficients of this matrix can be considered as a variable polynomial of the third degree, admitting three complex roots. Thus, the determination of *X_i_* is obtained by choosing the minimum (min) of the real part (real) among the roots (roots) of the one-line matrix Mi. This leads to the formula (46) below:

(46) $$X_{i}=\min\left(real\left(roots\left(M_{7}\right)\right)\right)$$

The determination of NH_3_ mass fraction of NH_3_-NaSCN solution *X_i_* associated with the laws of conservation of mass, chemical species and energy and then, the knowledge of the efficiency *ef* of the heat exchanger and the temperatures *T_i_* of the different components, allows to determine the enthalpy, the mass flow and the pressure at each point i (1≤i≤10) of the system.Thus, by replacing the NH_3_-NaSCN solution *X_i_* in (3), (6), (9), (12) we obtain the new models of ${\dot{Q}}_{g}$, ${\dot{Q}}_{e}$, ${\dot{Q}}_{c}$, and ${\dot{Q}}_{a}$ depending on the characteristic matrix *M_i_* respectively. These newly obtained models are given by Eqs. (47)–(50) below:

(47) $${\dot{Q}}_{g}=\left(\frac{min\left(real\left(roots\left(M_{2}\right)\right)\right)-min\left(real\left(roots\left(M_{8}\right)\right)\right)}{1-min\left(meal\left(roots\left(M_{8}\right)\right)\right)}h_{3}+\frac{1-min\left(real\left(roots\left(M_{2}\right)\right)\right)}{1-min\left(meal\left(roots\left(M_{8}\right)\right)\right)}h_{8}-h_{2}\right){\dot{m}}_{2}$$

(48) $${\dot{Q}}_{e}=\left(\frac{min\left(real\left(roots\left(M_{2}\right)\right)\right)-min\left(real\left(roots\left(M_{8}\right)\right)\right)}{1-min\left(real\left(roots\left(M_{8}\right)\right)\right)}{\dot{m}}_{2}\right)\left(h_{5}-h_{6}\right)$$

(49) $${\dot{Q}}_{c}=\left(\frac{min\left(real\left(roots\left(M_{2}\right)\right)\right)-min\left(real\left(roots\left(M_{8}\right)\right)\right)}{1-min\left(real\left(roots\left(M_{8}\right)\right)\right)}{\dot{m}}_{2}\right)\left(h_{3}-h_{4}\right)$$

(50) $${\dot{Q}}_{a}=\left(\frac{\mathrm{Min}\left(\mathrm{Real}\left(\mathrm{Roots}\left(R_{2}\right)\right)\right)-\mathrm{Min}\left(\mathrm{Real}\left(\mathrm{Roots}\left(M_{8}\right)\right)\right)}{1-\mathrm{Min}\left(\mathrm{Real}\left(\mathrm{Roots}\left(M_{8}\right)\right)\right)}h_{6}+\frac{1-\mathrm{Min}\left(\mathrm{Real}\left(\mathrm{Roots}\left(M_{2}\right)\right)\right)}{1-\mathrm{Min}\left(\mathrm{Real}\left(\mathrm{Roots}\left(M_{8}\right)\right)\right)}h_{10}-h_{7}\right){\dot{m}}_{2}$$

Replacing (15), (47) and (48) respectively in (42), (43) and (44) gives the CR, COP and ECOP respectively as a function of *M_i_*, hence Eqs. (51), (52) and (53) below:

(51) $$\text{CR}=\frac{1-min\left(real\left(roots\left(M_{8}\right)\right)\right)}{min\left(real\left(roots\left(M_{2}\right)\right)\right)-min\left(real\left(roots\left(M_{8}\right)\right)\right)}$$

(52) $$\text{COP}=\frac{\left(\frac{min\left(real\left(roots\left(M_{2}\right)\right)\right)-min\left(real\left(roots\left(M_{8}\right)\right)\right)}{1-min\left(real\left(roots\left(M_{8}\right)\right)\right)}{\dot{m}}_{2}\right)\left(h_{5}-h_{6}\right)}{\left(\frac{min\left(real\left(roots\left(M_{2}\right)\right)\right)-min\left(real\left(roots\left(M_{8}\right)\right)\right)}{1-min\left(real\left(roots\left(M_{8}\right)\right)\right)}h_{3}+\frac{1-min\left(real\left(roots\left(M_{2}\right)\right)\right)}{1-min\left(real\left(roots\left(M_{8}\right)\right)\right)}h_{8}-h_{2}\right){\dot{m}}_{2}+\frac{{\dot{m}}_{1}}{\rho }\left(P_{1}-P_{7}\right)}$$

(53) $$ECOP=\frac{\left(\frac{\min\left(real\left(roots\left(M_{2}\right)\right)\right)-\min\left(real\left(roots\left(M_{8}\right)\right)\right)}{1-\min\left(real\left(roots\left(M_{8}\right)\right)\right)}{\dot{m}}_{2}\right)\left(h_{5}-h_{6}\right)\left(1-\frac{T_{0}}{T_{e}}\right)}{\left(\frac{\min\left(real\left(roots\left(M_{2}\right)\right)\right)-\min\left(real\left(oots\left(M_{8}\right)\right)\right)}{1-\min\left(real\left(roots\left(M_{8}\right)\right)\right)}h_{3}+\frac{1-\min\left(real\left(roots\left(M_{2}\right)\right)\right)}{1-\min\left(real\left(roots\left(M_{8}\right)\right)\right)}h_{8}-h_{2}\right){\dot{m}}_{2}\left(1-\frac{T_{0}}{T_{g}}\right)+\frac{{\dot{m}}_{1}}{\rho }\left(P_{1}-P_{7}\right)}$$

### Algorithmic details

**Fig. 2:**
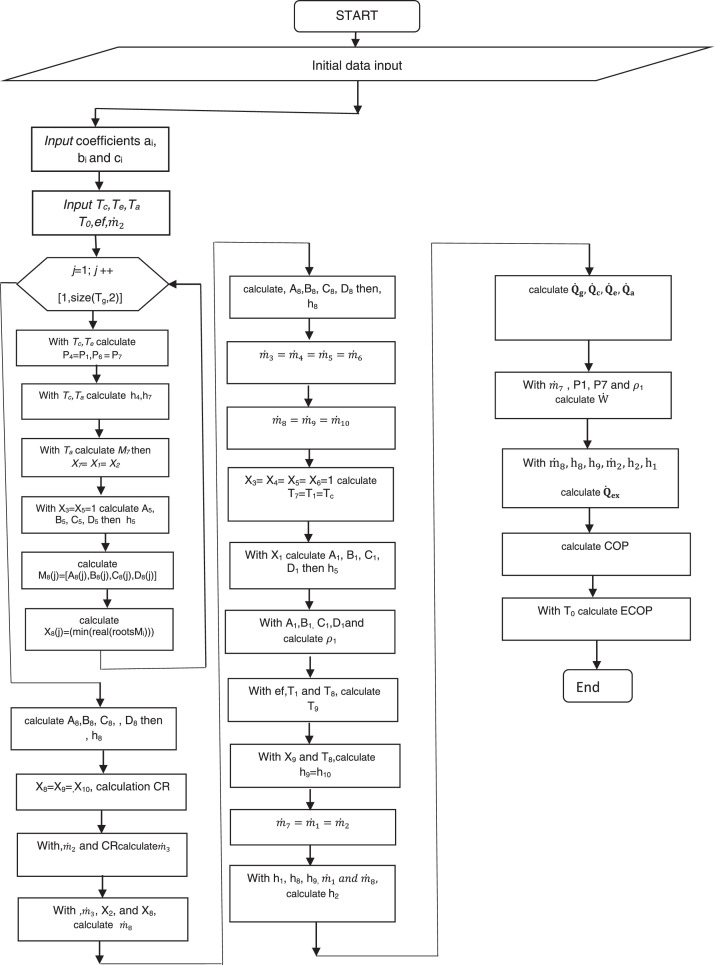
Design flowchart.The above algorithm is interpreted numerically by a fast, objective scientific program, using simple code.

The model developed above is a set of inter related equations. In reality, this overall model can be seen as a multivariate function whose solution depends on the objective. To start solving it, we first have to initialise some reference parameters. Each researcher is free to choose his or her own parameters to initialise, depending on the operating conditions of the cycle. After the initialization step, we have to calculate at each point i of the system (1≤*i*≤10), all the parameters needed for the targeted objectives. The last step consists in studying in turn the behaviour of each objective parameter (COP, ECOP, CR), as a function of any parameter of said system. For this purpose, we vary the latter, assigning reasonable values to the rest of the parameters in order to observe the variation of a characteristic performance parameter. However, as the generator is one of the main components at the start of the operation, it would be necessary to establish a detailed and reproducible flowchart based on the temperature variations of this unit. The same approach can be applied by varying the temperatures of the condenser, evaporator and absorber one after the other. The algorithm for solving the model equations is shown in the Fig. 2 below:

**Fig. 3:**
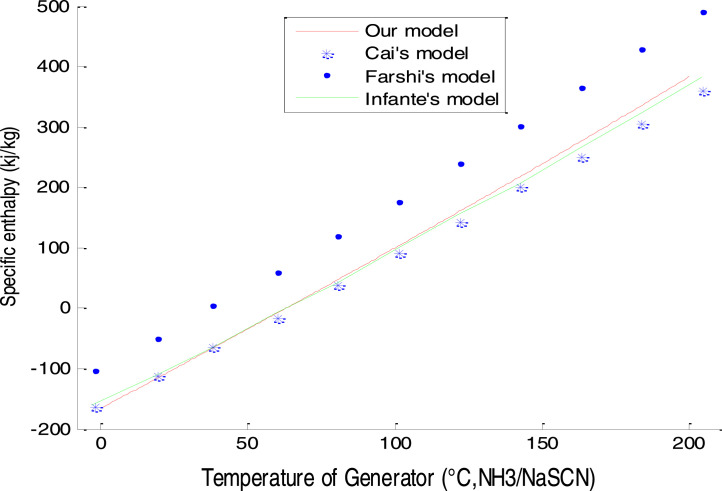
Comparison of our enthalpy model for XNH3=0.425.

## Validation of the model

The validation of a model is the direct confrontation of the results found against, those obtained with other researchers [5]. For this purpose, we have developed a scientific program which calculates the thermodynamic properties at each point of the system. The performance parameters from our model were compared with the original published data.

However, the original model of this work is based on the methodology of Sun [1] which does not take into account the entropy and therefore, the internal dissipation losses are not considered in the proposed method. Furthermore, there are other reference models in the literature that have used the same correlations as the original model of this work but, considering internal dissipation losses. One such example is the model developed by Zu and Gu [2]. For this reason, it may be necessary to make a comparison with the method proposed by Zu and Gu [2], although it is not the main original method used in this work. This may allow some analysis and conclusions to be drawn due to the limitations of not considering internal dissipation losses.

Furthermore, it is found that the expressions of COP and ECOP of the proposed model are also expressed depending on the enthalpy on different points. It would be necessary to validate the enthalpy model developed in this work with other models established by other researchers in the literature. This can anticipate a future consolidation of the COP and ECOP of the present model. However, the original model presented above does not take entropy into account, so the internal dissipation losses are not considered in the proposed model. Thus, a validation of the COP in line with the original model could also imply a validation of the ECOP, due to its expression including the constant terms $\left(1-\frac{T_{0}}{T_{e}}\right)$ et $\left(1-\frac{T_{0}}{T_{g}}\right)$.

### Enthalpy validation

The enthalpy is an intermediate and relational parameter to the performance parameters sought (COP ECOP, CR). This is a partial consolidation result of our model. The comparison of this result with Cai et al [6] is presented as follows in Fig. 3.

From this comparison with other researchers, we see that the present model is much closer to Ferreira's model. However, it does not confirm full validation because it does not take into account several parameters: it is a result of encouraging the correctness of the start of a scientific computing program.

### Validation of the Coefficient of Performance

**Fig. 4:**
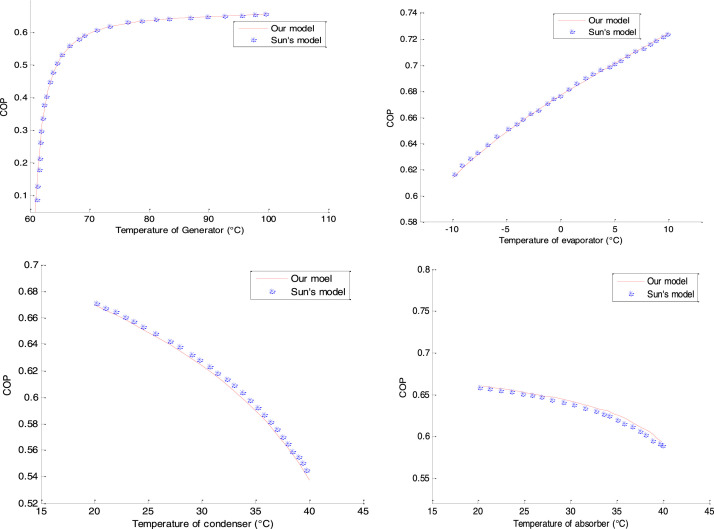
Validation of the Coefficient of Performance.

To validate our COP, we introduce the data from Sun [1] into our model and then compare the results of the different COP models from the generator, evaporator, condenser and absorber. For a temperature variation inside a component, the reference values of Sun [1] are: T_g_=90 °C, T_e_=−5 °C, T_c_=25 °C, T_a_=25 °C. Thus, we obtain the following confrontations as presented in Fig. 4:

The different Coefficients of Performance resulting from the generator, evaporator, condenser and absorber respectively show good agreement, and good agreement was found with the results of Sun [1].

### Validation of the circulation ratio

**Fig. 5:**
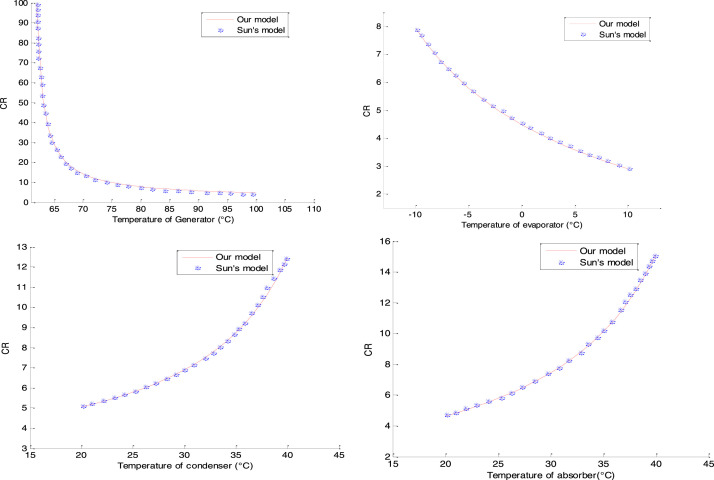
Validation of the circulation rate.

We compare our circulation ratio with that of sun [1] in the generator, evaporator, condenser and absorber respectively. The curves illustrated in Fig. 5 show the validation in the different components.

The different circulation rate results obtained in the generator, evaporator, condenser and absorber are also in agreement with the reference results

### Validation of exergy performance

Table 2 shows the results of the comparison of the different powers of the system components, and a good agreement was observed.

**Table 2:** Comparison of power in each component.

Components	Powers	Results of sun	Our results	Relative Error %
Generator	${\dot{Q}}_{g}$ (w)	29.0292	29.0304	0.0041
Evaporator	${\dot{Q}}_{e}$ (w)	18.5974	18.5617	0.1919
Condenser	${\dot{Q}}_{c}$ (w)	18.4611	18.5106	0.2681
Absorber	${\dot{Q}}_{a}$ (w)	29.2425	29.167	0.2581
Pump	$\dot{W}$ (w)	0.0771	0.0768	0.3891

Since in the already validated COP model expression, the respective heat out puts in the generator and evaporator are multiplied by the constant factors: $\left(1-\frac{T_{0}}{T_{e}}\right)$ and $\left(1-\frac{T_{0}}{T_{g}}\right)$. This consolidates the accuracy of our ECOP model.

### Limitations of the method and comparison

**Fig. 6:**
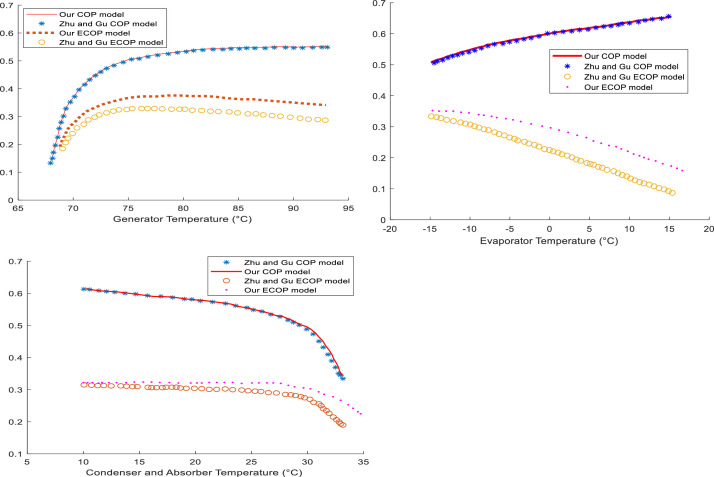
Comparison with the mathematical formulation of Zhu's model [2].

The method developed in this work does not allow the determination of internal losses in the different components of the present system. This is due to the absence of the mathematical formulation of entropy in our model. However, ECOP is the most exergy-significant of all the performance criteria [7]. Moreover, there is a mathematical formulation of ECOP closer to reality that includes entropy: this is the case formulated by Zhu and Gu [2]. Thus, the ECOP model developed in this work only takes into account the losses due to the temperature difference. The comparison of the present model with that of Zhu and Gu [2] is presented in Fig. 6.

From this comparison, it can be suggested that our mathematical formulation of the COP is similar to that of the reference, which is why the COP curves are almost identical. However, the ECOP obtained in each component has the same shape; but deviations from the original curves are observed. There are several reasons for these deviations. Indeed, Ghu and Zhu [2] considered the internal dissipation losses whereas in this method, we only consider the losses due to the temperature difference. This is an error due to the mathematical formulation because we have simplified the problem.

## Declaration of Competing Interest

The authors declare no conflicts of interest. In addition, this research did not receive any specific grant from funding agencies in the public, commercial, or not-for-profit sectors.

## Data Availability

No data was used for the research described in the article.
